# A rare case of volvulus after off-pump coronary artery bypass graft surgery in an opium addict patient revised

**DOI:** 10.22088/cjim.12.0.417

**Published:** 2021

**Authors:** Feridon Sabzi, Mohammad Rozbahani, Aghighe Heidar, Reza Faraji

**Affiliations:** 1 Kermanshah Cardiovascular Research Centre, Kermanshah University of Medical Sciences, Kermanshah, Iran; 2 Yazd Cardiovascular Research Center, Shahid Sadoughi University of Medical Sciences, Yazd, Iran

**Keywords:** Volvulus, Colonoscopy, Laparatomy.=

## Abstract

**Background:**

Volvulus of colon is a very rare phenomenon in post cardiac surgery course, and their predicting factor in most patients is unknown. Between colonic volvulus, splenic flexure is the rarest site for torsion in general population. The main symptoms are vague abdominal pain, vomiting and distension. The primary diagnostic images include plain chest x-ray, CT scan and colonoscopy.

**Case Presentation:**

We report the case of a 57-year -old opium male addict, who was. Admitted for abdominal pain, nausea, and vomiting five days after off-pump coronary artery bypass surgery (OPCAB). An abdominal x-ray reported a colonic volvulus. Exploratory laparotomy showed acute abdomen resulting from a gangrene of long segment of splenic flexure caused by volvulus.

**Conclusion:**

Gastrointestinal complication such as volvulus is an exceedingly rare complication of OPCAB, despite the absence of anatomic abnormalities only complete colonic malrotation as the result of mega colon and constipation, the main pathogenetic causes. This patient was unique because of careful literature search revealed that this case was the first reported volvulus that has been described so far.

Splenic colon flexure is a rare site for colonic volvulus and its incidence is even exceedingly rare in OPCAB. However, cardio pulmonary bypass (CPB) is excluded from surgery in OPCAB, but in addicted patients, a specific syndrome named as narcotic bowel syndrome has been reported (1). In many nonsurgical cases, the incomplete torsion caused incomplete obstruction and recurrent abdominal pain and discomfort. The most common place for colonic volvulus is sigmoid colon followed by caecum. Other rare sites of volulus including transverse colon and the rarest type is splenic flexure. The sign and symptoms of volvulus are similar to colonic obstruction. Ct scan is the main diagnostic imaging method however, diagnosis may be missed in postoperative surgical cases due to anesthesia and incision pain. However, anatomic defect is the main cause of volvulus, but severe constipation may be a rare predisposing factor in postsurgical course. However, constipation is not uncommon in postoperative course but oral opium use is the main cause of severe constipation. Most of the patients are opium smokers but surgical patients have to use the opium orally that led to constipation. However, it does not infiltrate the blood-brain dam, opium increases the volume and density of internal opioid peptides at opioid recipients in the gastro intestinal canal (1), the use of oral opium in postoperative period in an opium smoker is accompanied with a number of side effects among which constipation and volvulus are the most troublesome. 

The splenic flexure is a rarest cause of volvulus in general population (2). The involved patients almost have determined risk factors for example previous abdominal laparatomy, retroperitoneal dissection involving releasing of the splenic flexure with consequent adherences, congenital lack of the ligamentous adhesions of the splenic flexure or immune disorders such as systemic lupus erythromatous, multiple sclerosis affecting the colon (3). The main diagnostic and therapeutic intervention includes colonoscopy that may detort the volvulus and treat the complication, on the other hand, if colonoscopy does not detort the volvulus, the next step will be laparatomy. In addition to colonoscopy, other diagnostic tools are simple abdominal x-ray and contrast, CT scan of abdomen.

## Case presentation 

Contrary to many efforts to expand other therapeutics, oral opium (taryak) remains as the basic remedy or auto therapy in many opium-addict patients with pain and to prevent withdrawal syndrome. This report shows a case of splenic flexure volvulus in an addict patient leading to a major gangrene and peritonitis.

**Figure 1:**
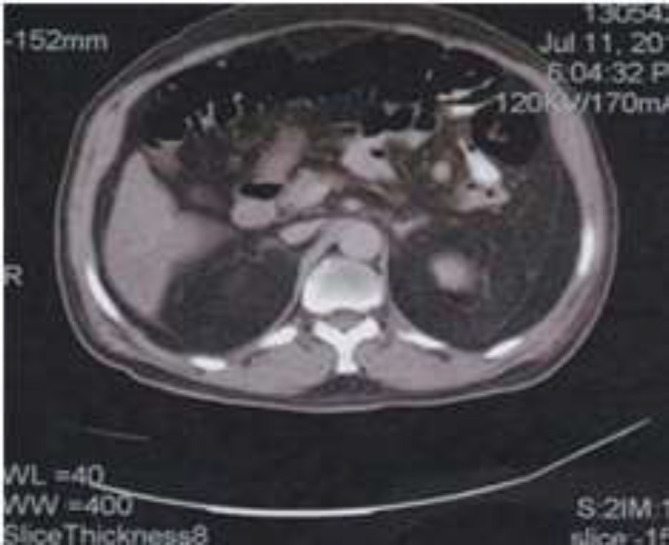
CT scan revealed distended transverse colon

**Figure 2:**
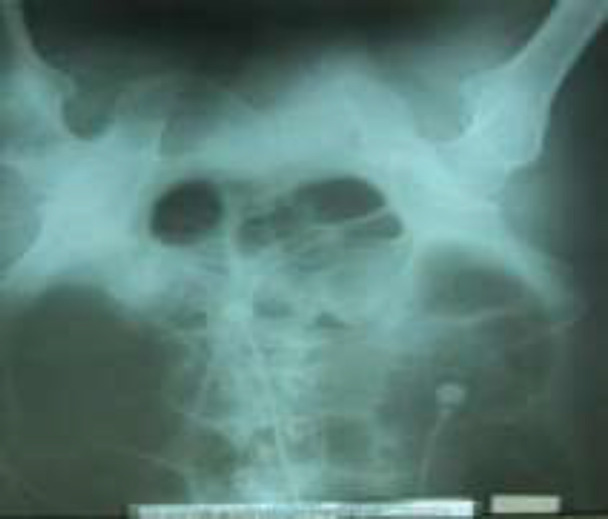
Revealed distended transverse colon

A 57-year-old man presented in Imam Ali Hospital in 2017 with unstable angina due to severe stenosis of the left anterior descending coronary artery and obtuse marginal, however, the right coronary artery was normal. Laboratory finding including a complete blood count and erythrocyte sedimentation rate and C reactive protein was normal. Chest- ray revealed no abnormal finding. There was not any valular abnormality by preoperative echocardiography. The patient has not had any co-morbid disorder. Left ventricular ejection fraction was reduced (45-40%). The intraoperative course was uneventful. There was no any finding with regard to pericarditis. On physical examination, patient was not in respiratory distress, blood pressure was 130/80 mmHg, heart rate 80 beat per minute, respiratory rate 23/min, neck vein was not distended and without ankle edema. On examination of the cardiovascular system, he had a regular s1 s2 with no gallop. No murmur was appreciated. ECG showed Q wave in precordial leads. The patient underwent uneventful off-pump coronary artery bypass grafting. There was not any finding regarding pericarditis and postoperative ESR and CRP were normal. After hemodynamic stabilization, the patient left the intensive care unit without IABP and inotropic support. The patient presented with distension without nausea and vomiting in the 3rd postoperative day. His past medical history included no history of abdominal pain episodes with abdominal distension, or laparatomy or immune disease, in surgical consultation with an expertise general surgeon, a postoperative ileus was diagnosed. Primary abdominal x-ray revealed only distension of colon. 72 hours after the first consultation, with increasing abdominal distension and poor general condition of patient, abdominal x-ray showed a clear distension of colon suggestive for a volvulus (figure 1). On this presentation, his abdomen was not tender despite massive distension confined mainly to the upper abdomen. Bowel sounds were present. Plain abdominal and chest radiographs revealed a markedly distended colon, and a single loop of dilated large intestine filling the entire abdomen (figure 2). 

Abdominal CT scan with contrast revealed distended transverse colon (figure 1). A colonoscopy showed the twist, apparently situated in the distal sigmoid colon. Despite repeated attempts, endoscopic decompression of the volvulus failed. Explorative laparotomy was therefore performed, revealing a clockwise 360° rotation of the splenic flexure. At laparotomy, the splenic flexure was found to be grossly dilated and had undergone a 180º counterclockwise rotation causing mechanical obstruction with evidence of gangrene. The dilated segment was resected and a primary anastomosis was performed between the transverse and descending colon. Subsequent hospitalization was uneventful and the patient was discharged 8 days after. Up to now, the patient has not experienced any recurrence.

## Discussion

The mechanism of splenic flexure volvulus in this specific case has been attributed to displacement, compression and partial obstruction of flexure colon by the chronic constipation. Diagnosis of the condition due to illicit opium use and its analgesic effect is often delayed. The abdominal pain is absent primarily in this patient. The average length of time from the onset of obstructive symptoms until presentation in this patients is 48 hours, because oral opium itself clouds the clinical picture such abdominal pain, nausea, however distension can occur in an otherwise postsurgery periods.In Sever et al.‘s study, large series of patients (1360 adult cardiac surgery) 29 patients with mesenteric ischemia was reported but no case of volvulus has been found (4). In Viana et al.’s study, during 10 years period, no case of volvulus was reported (5). However, volvulus is not rare in general surgery but its incidence in post cardiac surgery is very rare and no case of splenic flexure volvulus was reported in medical literature. We believe that our patient is the first case of splenic flexure volvulus in medical literature. The first patient with a splenic flexure volvulus in general surgery was described by Glazer et al. in 1953 (6). 

In Raht et al.’s study volvulus was confined to the sigmoid (70%), but it may also affect the right (25%) and transverse colon (2-5%) (7). In Ballantyne’s study, splenic flexure volvulus was the cause of torsion for small numbers of colonic volvulus. Predisposing factors in general surgery are the congenital absence gastrocolic, phrenocolic, splenocolic ligament or surgical excision of gastrocolic, phrenocolic, splenocolic ligament (8). Atamanalp revealed that splenic colon volvulus has a poor outcome in comparison with other types (9). Hashemzadeh et al. described the method of colon imaging especially splenic flexcure volvulus (10). Halaby et al. showed that colonic flexure volvulus is a risk factor for postoperative moratlity (11). Yassaie et al. depicted that the early surgery is justifiable in volulus of colon (12). Bruzzi et al. reported that early outcome of surgery in volvulus increases the early outcome (13). When these factors are present, the splenic flexure will be associated with high mobility. The association of chronic constipation in opium-addict patients may lead to distention of the colon, a condition often associated with mesentery stretching and volvulus. This pathologic condition was exacerbated with oral opium in postoperative period. The diagnosis is clouded by vague clinical presentation, often represented by only distension and absence of pain. Splenic flexure volvulus is often diagnosed in the theatre as in our case report. When the bowel is viable, there are several choices: detorsion followed by elective surgery, exteriorization of splenic flexure, resection with primary or delayed anastomosis. Partial colectomy or exteriorization of the non-viable tract is mandatory when gangrene is present (14).

In conclusion, we think that mega colon caused by chronic constipation induced by oral opium had an important pathogenetic role to elicit the splenic flexure volvulus. Unique finding about this case is related to rarity of volvulus of splenic felcture in cardiac surgery and indeed it was the first case of splenic flexure volvulus that was reported in medical literature in off-pump CABG so far. 
